# Recombinant measles vaccine expressing malaria antigens induces long-term memory and protection in mice

**DOI:** 10.1038/s41541-019-0106-8

**Published:** 2019-02-27

**Authors:** Marie Mura, Claude Ruffié, Chantal Combredet, Eduardo Aliprandini, Pauline Formaglio, Chetan E. Chitnis, Rogerio Amino, Frédéric Tangy

**Affiliations:** 1 Viral Genomics and Vaccination, Institut Pasteur, CNRS UMR-3569, 28 rue du Dr Roux, 75015 Paris, France; 2 Anti-infectious Biotherapies and Immunity, Institut de Recherche Biomédicale des Armées, 1 place du Général Valérie André, BP73 Brétigny-sur-Orge Cedex, France; 3 Malaria Infection and Immunity, Institut Pasteur, CNRS UMR-3569, 28 rue du Dr Roux, 75015 Paris, France; 4 Malaria Parasite Biology and Vaccines, Institut Pasteur, CNRS UMR-3569, 28 rue du Dr Roux, 75015 Paris, France

## Abstract

Following the RTS,S malaria vaccine, which showed only partial protection with short-term memory, there is strong support to develop second-generation malaria vaccines that yield higher efficacy with longer duration. The use of replicating viral vectors to deliver subunit vaccines is of great interest due to their capacity to induce efficient cellular immune responses and long-term memory. The measles vaccine virus offers an efficient and safe live viral vector that could easily be implemented in the field. Here, we produced recombinant measles viruses (rMV) expressing malaria “gold standard” circumsporozoïte antigen (CS) of *Plasmodium berghei* (*Pb*) and *Plasmodium falciparum* (*Pf*) to test proof of concept of this delivery strategy. Immunization with rMV expressing *Pb*CS or *Pf*CS induced high antibody responses in mice that did not decrease for at least 22 weeks post-prime, as well as rapid development of cellular immune responses. The observed long-term memory response is key for development of second-generation malaria vaccines. Sterile protection was achieved in 33% of immunized mice, as usually observed with the CS antigen, and all other immunized animals were clinically protected from severe and lethal *Pb* ANKA-induced cerebral malaria. Further rMV-vectored malaria vaccine candidates expressing additional pre-erythrocytic and blood-stage antigens in combination with rMV expressing *Pf*CS may provide a path to development of next generation malaria vaccines with higher efficacy.

## Malaria: Recombinant measles virus malaria vaccine

Following the limited success of the RTS,S recombinant malaria vaccine there is a pressing need for second generation malaria vaccines. Frédéric Tangy and colleagues at the Pasteur Institute, Paris, generate novel vaccines based on recombinant measles virus (rMV) expressing the major circumsporozoite antigen CS from either *Plasmodium berghei* (rMV-CS*Pb*) or *P. falciparum* (rMV-CS*Pf*). rMV is a strong vector candidate because of its widespread use, safety profile and efficacy. Mice permissive to rMV infection show rapid and durable (at least 22 weeks) CS antibody responses as well as activation of cell-mediated immunity and type 1 helper responses following vaccination with rMV-CS*Pb* or rMV-CS*Pf*. rMV-CS*Pb* vaccination protects mice from lethal challenge with *Pb* sporozoites, and in a subset of mice leads to sterile immunity. The rMV vector offers the potential of incorporating further antigens from other *Plasmodium* infection stages and thereby enhancement of vaccine efficacy.

## Introduction

Despite decades of malaria vaccine research, only RTS,S/AS01 vaccine candidate reached Phase III clinical trial to eventually show moderate protection of short duration.^1^ This led the World Health Organization to recommend additional pilot studies in three countries with enhanced pharmacovigilance.^2^ Nevertheless, these results are encouraging as they establish the feasibility of developing a malaria vaccine. Furthermore, the spread of artemisinin-resistant *P. falciparum* strains^3^ underlines the need for an effective vaccine for sustained protection against malaria. The rationale for malaria vaccine development relies on several observations. First, natural immunity is gradually acquired to severe, life-threatening malaria and then to clinical disease after several years of natural exposure.^4^ Nevertheless, this immunity is not sterile and quickly wanes if an individual leaves the endemic area. Continued exposure to parasites is, therefore, required to maintain immunological memory.^5^ Second, transfer of gamma-globulin fractions from semi-immune to naïve humans clears blood stage parasites and mitigates malaria disease.^6^ Finally, inoculation of irradiated attenuated sporozoïtes can protect humans against infectious challenge, but requires high and frequent doses, and immunity wanes after 6 months.^7^ Therefore, the induction of long-term memory is critical for sustained vaccine efficacy.

The RTS,S subunit vaccine is based on the *Plasmodium falciparum* (*Pf*) circumsporozoïte protein (CS), which is expressed during the sporozoïte and early liver stages, and is involved in adhesion and invasion of hepatocytes. CS is known as the lead antigen for inclusion in a pre-erythrocytic vaccine candidate. Based on data on efficacy elicited by CS in pre-clinical as well as human challenge models, the CS is considered a “gold standard” that can be used to evaluate different vaccine delivery platforms and prime-boost strategies.^8–10^ The CS is composed of a central and conserved Asparagine-Alanine-Asparagine-Proline (NANP) amino acid repeat sequence, known as the immunodominant B-cell epitope. Indeed, CS-specific antibodies and CD4^+^ T cell responses were associated with human protection during RTS,S-controlled human malaria infection trials (CHMI).^11^ However, RTS,S/AS01 did not induce CD8^+^ T cell responses, which play an important role in parasite elimination in the liver.^12^ Viral vectors are known for their capacity to induce CD8^+^ T cell response but prime-boost strategies with AdCh63 and MVA, which are non-replicative viral vectors, were disappointing.^13,14^

The measles virus (MV) vector-based vaccine platform offers new opportunities as a replicative but safe viral vector. It has not yet been used to deliver malaria antigens. The rationale for the use of MV is based on the following arguments: (i) MV is one of the safest and most effective human vaccines, eliciting life-long protective immunity against measles after a single injection; (ii) its production can be easily scaled up at low cost, which is important for developing countries where malaria is endemic; (iii) immunization with MV vector induces both humoral and cellular responses to the transgenes^15–19^; (iv) MV genome can integrate up to 6 kb in additional transcription units, allowing the expression of several malaria antigens; (v) phase I and phase II clinical trials with a recombinant MV (rMV) vaccine expressing chikungunya virus-like particles showed that the vaccine was very immunogenic and, unlike non-replicative viral vector platforms, there was no impact of pre-existing immunity against measles vector^20,21^; (vi) In 2016, about 85% of the world’s children received one dose of measles vaccine by their first birthday through routine health services. A recombinant measles-malaria vaccine could therefore easily be integrated in vaccination schedules.

Based on previously obtained data in collaboration with R. and V. Nussensweig, we have generated rMV expressing the CS protein of *Plasmodium berghei* (*Pb*) and *Pf* to establish proof of concept for the use of measles vector to express malaria antigens. In the CS*Pb* model, we demonstrate that rMV-CS*Pb* is able to induce sterile protection of mice or at least protect them from severe symptoms with reduced blood parasitemia. In the CS*Pf* model, rMV-CS*Pf* induced immunogenicity has a Th1 profile and is maintained from 3 weeks up to, at least, 4 months after the second immunization. Furthermore, we show the induction of CD4^+^ and CD8^+^ cellular responses. High antibody titers with long-term memory and induction of cellular response are keys for the development of a malaria vaccine with higher efficacy and long-term protection against *P. falciparum* malaria.

## Results

### Production of rMVs expressing CS*Pb* and CS*Pf* proteins

**Fig. 1:**
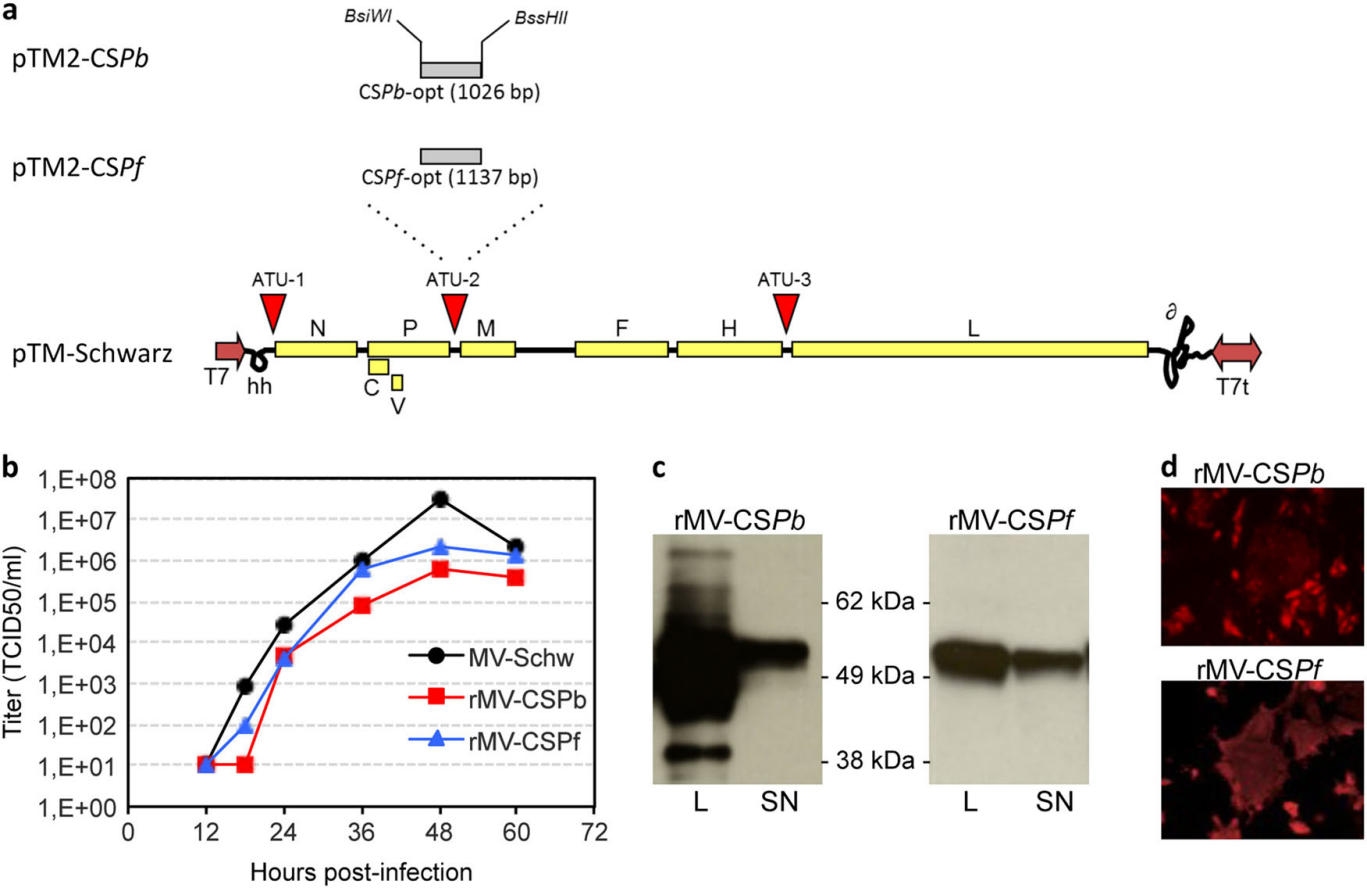
rMV-CS*Pb* and rMV CS*Pf* design, rescue and characterization. **a** Schematic representation of measles vector expressing CS protein from *Plasmodium berghei* ANKA (CS*Pb*) and *Plasmodium falciparum* (CS*Pf*). The synthetic sequences were mammalian codon-optimized and cloned into the additional transcription unit (ATU) position 2 of pTM-Schwarz. The MV genes are indicated as follows: nucleoprotein (N), phosphoprotein (P), V and C accessory proteins, matrix (M), fusion (F), hemagglutinin (H) and polymerase (L). T7 RNA polymerase promoter (T7), T7 RNA polymerase terminator (T7t), hepatitis delta virus ribozyme (∂), hammerhead ribozyme (hh) are requested for viral rescue. **b** Growth curves of MV-Schwarz, rMV-CS*Pb*, and rMV-CS*Pf* in Vero cells infected at an MOI of 0.1. Cell-associated virus titers are indicated in TCID_50_/ml. **c** Detection by western-blot of CS*Pb* and CS*Pf* in cell lysates (L) or supernatant (SN) of Vero cells infected by rMV-CS*Pb* and rMV CS*Pf*. Blots derived from the same experiment and were processed in parallel**. d** Immunofluorescence detection of CS*Pb* and CS*Pf* in Vero cells infected for 24 h with rMV-CS*Pb* and rMV-CS*Pf* at an MOI of 0.1. Infected cells formed syncytia which are localized CS proteins

We constructed an rMV expressing CS*Pb* protein (rMV-CS*Pb*) and an rMV expressing CS*Pf* protein (rMV-CS*Pf*) by inserting mammalian codon-optimized sequences of both proteins in additional transcription unit 2 (ATU2) of pTM-MVSchw plasmid, which encodes the antigenome of the Schwarz MV vaccine strain^22^ (Fig. 1a). The ATU2 allows high-level expression of the protein, as there is a decreasing gradient of gene expression generated by MV replication (from high nucleoprotein “N” expression to low polymerase “L” expression). Both plasmids were transfected into HEK293T-helper cells for rescue and co-cultured with Vero cells for virus spread. The rescued rMV-CS*Pb* and rMV-CS*Pf* had slightly delayed growth curves, as compared to empty MV (Fig. 1b), but still reached high titers on Vero cells. Viral stocks were made from unique syncytia after rescue and are therefore considered as clonal. The expression of CS was assessed by Western blot, and found in the lysate and in the supernatant of infected Vero cells (Fig. 1c and Supplementary Fig. 1). The CS expression in infected cells forming syncytia was also demonstrated by immunofluorescence (Fig. 1d). For rMV-CS*Pf*, the stability of transgene expression was demonstrated after 10 passages of the recombinant virus on Vero cells by immunofluorescence, Western blot and sequencing. The stability of rMV-CS*Pb* was not tested as the mouse model was only used for proof of concept but the virus stock was successfully characterized by sequencing and CS*Pb* expression analysis.

### Susceptibility of hCD46IFNAR mice to *Pb* ANKA challenge

**Fig. 2:**
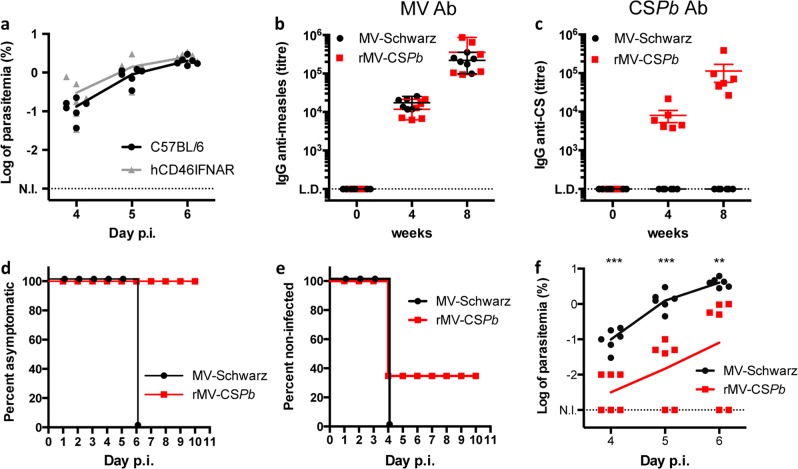
Immunogenicity and protective efficacy of rMV-CS*Pb*. **a** Blood parasitemia of C57BL/6 and hCD46IFNAR mice after skin microinjection of 5000 sporozoites of *Plasmodium berghei* ANKA. Percentage of infected red blood cells (iRBCs) at day 4, 5, and 6 post-infection (p.i.) was log transformed for parasitemia normalization before statistical analysis. No statistically significant difference was observed between both groups. **b**, **c** Antibody response induced in hCD46IFNAR mice immunized with rMV-CS*Pb* at day 0 and 4 weeks later. The data show the reciprocal endpoint dilution titers of specific antibodies to MV **b** and CS*Pb* **c**. Percentage of asymptomatic **d** and non-infected **e** hCD46IFNAR mice (6 mice per group) immunized twice at one month of interval and challenged 3 weeks after with 5000 *Pb* ANKA sporozoïtes intradermally. **f** Log of parasitemia at day 4, 5 and 6 post-infection (p.i.) (lines represent means for each group). Asterisks (*) indicate significant mean differences (**p < 0.01; ***p < 0.001) measured by *t*-test after normalization. L.D. level of detection. N.I. threshold of parasitemia detection

Mice are naturally resistant to MV, which is restricted to human and non-human primates (NHPs). The usual mouse model to test rMV vaccine candidates is deficient for type-I IFN receptor (IFNAR) and expresses human receptor CD46 (hCD46).^22^ The genetic background of hCD46IFNAR mouse used here is Sv129, which has the same major histocompatibility complex haplotype as C57BL/6 mouse (H-2Db, H-2Kb, I-Ab). C57BL/6 mice infected with *P. berghei* ANKA (*Pb*A) is a model for cerebral malaria, which leads to death. C57BL/6 mice are easily infected and highly susceptible, as compared to Balb/c mice.^23,24^ In order to validate the model of infection in hCD46IFNAR mice, we inoculated 5000 GFP-expressing *Pb*A (GFP *Pb*A) sporozoites in the footpad of six C57BL/6 and six hCD46IFNAR mice. We monitored the parasitemia and clinical symptoms from day 4 to day 6 post-inoculation. Although parasitemia was slightly higher in hCD46IFNAR group, there was no statistically significant difference between both groups of mice (Fig. 2a). So, we concluded that both mouse models were comparable for sporozoite challenge. These observations validated the use of hCD46IFNAR mouse for the rest of the study.

### Immunogenicity and protective efficacy of rMV-CS*Pb* as a proof of concept

Six-week-old hCD46IFNAR mice (6 mice per group) received 10^5^ TCID_50_ of rMV-CS*Pb*, or MVSchw as negative control, by intraperitoneal (i.p.) route at day 0 and at day 28. Sera were collected before the first immunization (control) and 3 weeks after each immunization. Antibodies to MV were induced at similar levels in all immunized mice (Fig. 2b). Antibodies to CS*Pb* were efficiently induced from the first immunization with limiting dilution titers of about 10^4^, then boosted after the second immunization to reach 10^5^ (Fig. 2c). Mice were challenged 3 weeks after the second immunization with 5000 sporozoïtes of GFP-P*b*A injected in the footpad. In MVSchw immunized group (control), we sacrificed mice at day 6 post-challenge (Fig. 2d), due to start of cerebral symptoms, which were ethical endpoints of the study. In rMV-CS*Pb* immunized group, two mice (33%) achieved sterile protection (no detectable iRBC at day 10 post-challenge) and parasitemia was delayed for one mouse (Fig. 2e). The other mice showed a significant decreased parasitemia (Fig. 2f), with no observed severe symptoms. Moreover, at day 10 post-challenge, the parasitemia in rMV-CSPb immunized mice was still <1%. So, immunization with rMV-CS*Pb* achieved sterile protection in 33% of hCD46IFNAR mice and completely protected mice from severe and lethal P*b*A-induced cerebral malaria.

### Immunogenicity of rMV-CS*Pf*: Th1 IgG subtype profile and long-term memory

**Fig. 3:**
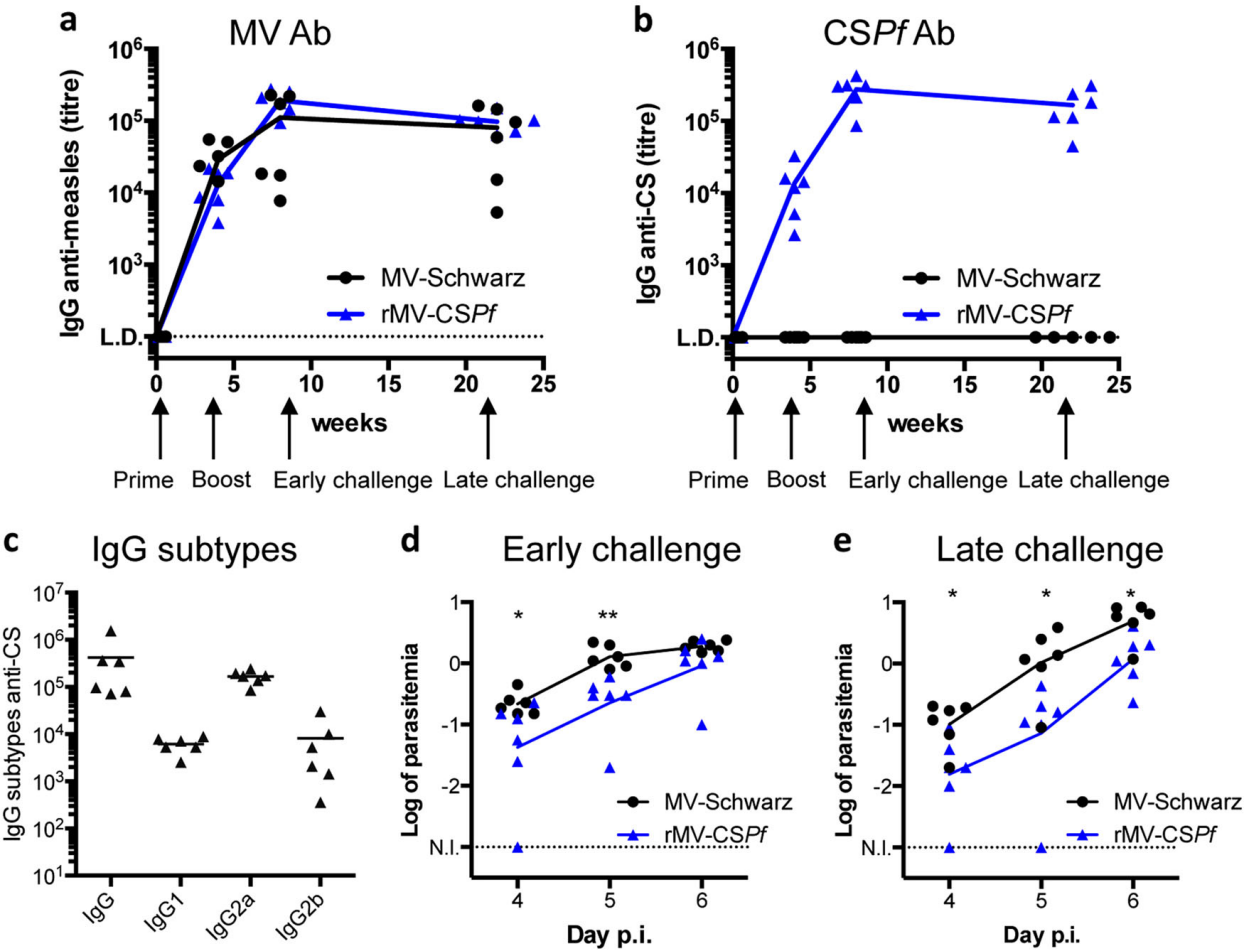
Immunogenicity and protective efficacy of rMV-CS*Pf* in hCD46IFNAR mice. **a**–**c** Antibody response induced in hCD46IFNAR mice immunized with rMV-CS*Pf* at day 0 and 4 weeks later. Long-term memory was assessed at week 22 post-priming. The data show the reciprocal endpoint dilution titers of specific antibodies to MV **a** and CS*Pf* **b**. **c** IgG subtypes of CS*Pf* antibodies elicited by rMV-CS*Pf* 4 weeks after the second immunization. **d**, **e** Infectious challenge with 5000 sporozoïtes of a recombinant *Pb*A expressing CS*Pf* repeat sequence 3 weeks **d** or 16 weeks **e** after the second immunization. Log of parasitemia at days 4, 5, and 6 post-infection (p.i.) (lines represent means for each group). Asterisks (*) indicate significant mean differences (*p < 0.05; **p < 0.01) measured by *t*-test after normalization. L.D. level of detection. N.I. threshold of parasitemia detection

Six-week-old hCD46IFNAR mice (6 mice per group) received 10^5^ TCID_50_ of rMV-CS*Pf*, or MVSchw as negative control, by i.p. route at day 0 and at day 28. Sera were collected before the first immunization (control), 3 weeks after each immunization, and 22 weeks after the first immunization for a group of 6 mice dedicated to long-term memory study. As for rMV-CS*Pb*, antibodies to MV were induced at similar levels in all immunized mice (Fig. 3a) and antibodies to CS*Pf* were efficiently induced from the first immunization with limiting dilution titers of about 10^4^, then boosted after the second immunization to reach 10^5^ (Fig. 3b). Interestingly, this high antibody titer was maintained 22 weeks post-prime. The humoral response profile corresponded to Th1 polarization with high titers of IgG2a antibodies (Fig. 3c), as expected for a replicative viral vector. Mice were challenged 3 weeks after the second immunization (early challenge) or 22 weeks post-prime (late challenge) with 5000 sporozoites of recombinant GFP-P*b* expressing CS*Pb* with CS*Pf* repeat sequence (rGFP-*Pb*-CS*Pf* repeat), microinjected in the mouse footpad. In MVSchw immunized group (control), all mice were sacrificed at day 6 post-challenge, due to start of cerebral symptoms. In rMV-CS*Pf* immunized group, there was no induction of sterile protection, but a decreased and delayed parasitemia, whether for early (Fig. 3d) or late challenge (Fig. 3e). Mice started to present symptoms of cerebral malaria at day 7 and were sacrificed to avoid unnecessary suffering. This decreased parasitemia was therefore less important than the one observed for rMV-CS*Pb*. For both studies, there was no correlation between protection and anti-CS antibody titers. We hypothesized that the observed difference was due to the challenge model with rGFP-*PbA*-CS*Pf* repeat that allow only to study protection relying on neutralizing antibodies directed against the repeat sequence. We therefore evaluated the cellular response in the *Pf* model.

### Induction of specific cellular immune response

**Fig. 4:**
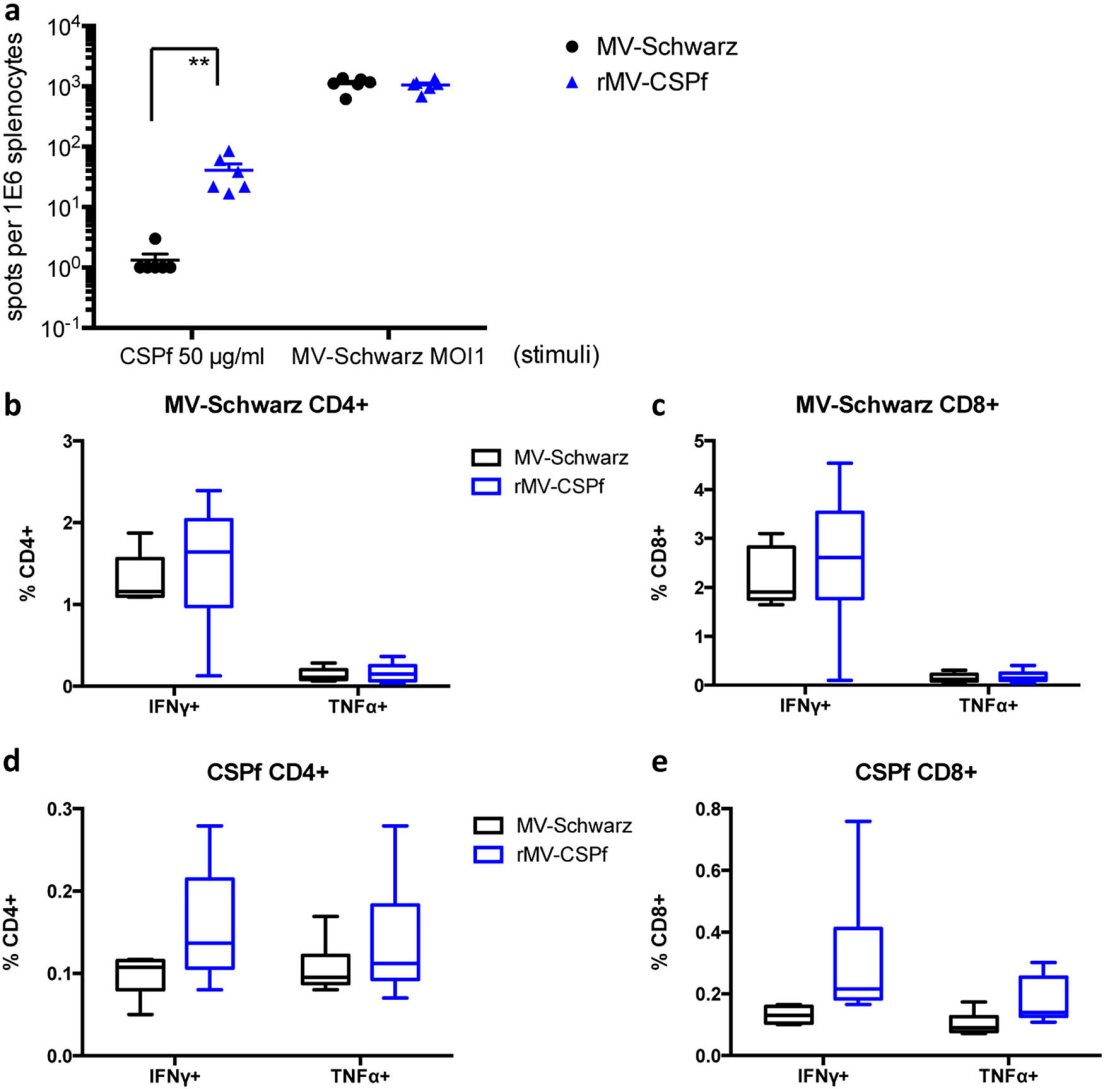
Cellular response to rMV-CS*Pf* in hCD46IFNAR mice. **a** IFNγ Elispot assay and **b** intracellular cytokine staining assay were done on freshly extracted splenocytes 7 days after one immunization i.p. with 1 × 10^5^ TCID_50_ of MV-Schwarz or rMV-CS*Pf*. Splenocytes were restimulated with inactivated MV-Schwarz at an MOI of 1 or CS*Pf* recombinant LPS-free protein at 50 µg/ml. CD4^+^ and CD8^+^ T-cells were stained against IFNγ and TNFα. Negative controls, cultured with media alone, showed <0.05% of positive cells. Percentile IFNγ and TNFα cytokine distribution (5th, 25th, 50th, 75th, and 95th percentiles) for CD4^+^ (left panel) and CD8^+^ (right panel) T-cells reactive against MV-Schwarz (**b1**) and upon CS*Pf* restimulation (**b2**). Asterisks (*) indicate significant mean differences (**p < 0.01) for Mann–Whitney *U*-test

Cell-mediating immune response (CMI) elicited by immunization with rMV-CS*Pf* was assessed using IFNγ Elispot assay and intracellular cytokine staining (IFNγ and TNFα) on freshly extracted splenocytes collected 7 days after a single immunization with 1 × 10^5^ TCID_50_ in 100 µl i.p. (Fig. 4 and Supplementary Fig. 2). Splenocytes were stimulated ex vivo with inactivated MV-Schwarz at an MOI of 1 or CS*Pf* recombinant LPS-free protein at 50 µg/ml. A moderate but significant (*p* < 0.01, Mann–Whitney *U* test) number of CS*Pf*-specific cells (up to 100/10^6^ splenocytes) were detected by the ELISPOT assay (Fig. 4a), which corresponds to 5–10% of the number of MV-specific spots. The phenotype of MV-specific and CS*Pf*-specific cells induced by rMV-CS*Pf* was analyzed by flow cytometry (Fig. 4b–e). The mean frequency of MV-specific T cells secreting IFNγ and TNFα in CD4^+^ cells (Fig. 4b) was, respectively, 1.5% and 0.2%. The mean frequency of MV-specific T cells secreting IFNγ and TNFα in CD8^+^ cells (Fig. 4c) was respectively, 2.6% and 0.2%. The mean frequency of CS*Pf*-specific T cells secreting IFNγ and TNFα in CD4^+^ cells (Fig. 4d) was respectively, 0.16% and 0.14%. The mean frequency of MV-specific T cells secreting IFNγ and TNFα in CD8^+^ cells (Fig. 4e) was respectively, 0.3% and 0.18%. An induction of CD4^+^ cells secreting IFNγ and CD8^+^ cells secreting IFNγ or TNFα was observed, as compared to control group but statistically not significant (*p* = 0.074, *p* = 0.057, and *p* = 0.088, respectively, Mann–Whitney *U*-test). Even if no CD8^+^ epitopes of CS*Pf* were described in C57BL/6 mouse, we showed the induction of a moderate but significant CMI as early as 7 days after a single immunization with rMV-CS*Pf*, with CD4^+^-activated and CD8^+^-activated phenotype.

## Discussion

Following the moderate protection and short memory response induced by RTS,S vaccine candidate in phase III clinical trial,^1^ there is strong support for developing a second-generation malaria vaccine with higher efficacy and longer duration of protection. Because of its central place in infant vaccine schedules all over the world, measles provides a promising viral vector to deliver malaria antigens, either as a single delivery platform or in a prime boost strategy. We report here the first use of measles-based vaccine platform to deliver CS malaria antigen as a proof of concept of the feasibility and advantages of this vector, in a murine model. Importantly, we showed induction of cellular response and long-term memory with high antibody titers. These are the two main characteristics required for second-generation malaria vaccine candidates.

We first showed the possibility of stably expressing a malaria antigen using the MV as a delivery vector. CS*Pb* and CS*Pf* sequences were successfully inserted in MV-Schwarz genome and stably maintained after 10 passages in Vero cell culture, without any mutation. Nevertheless, we were unable to rescue a virus with CS native sequence and therefore mammalian codon-optimized sequence is required. The *P. falciparum* genome is AT rich^25^ and polyA/polyU probably disturbed measles polymerase, either for replication or transcription. As MV-Schwarz vector is able to insert 6 kb in its additional transcription units, other antigens could be easily added to CS to improve vaccine efficacy.

Then we showed in the hCD46IFNAR mouse model the induction of high antibody titers that are maintained at least until 22 weeks post-prime in a two-immunization schedule with one-month interval. This maintenance of high antibody level was longer than the one observed with CS administered in a three doses regimen at 50 µg with complete Freund’s adjuvant in C57BL/6 mice,^26^ whereas rMV delivered only ng of heterologous antigens.^17^ R16HBsAg, a precursor of RTS,S, induced high level of antibodies in mice when administered with alum in a three dose regimen, but was not assessed more than 5 weeks after the last immunization.^27^ In monkeys, RTS,S/AS01B formulation has shown a rapid decrease of CS antibodies 8 weeks after each boost.^28^ The long-term persistence of neutralizing antibodies against heterologous antigens vectored by rMV has already been described for an rMV expressing HIV antigens in both mouse^16^ and NHP models.^19^ Thus, the observed maintenance of high anti-CS antibody level is promising regarding MV efficiency to induce life-long memory. IgG sub-types were predominantly IgG2a, which was expected for a replicating viral vector. This subclass is cytophilic in mice,^29^ with complement fixation and pathogen opsonization. Moreover, induction of cytophilic CS antibodies has been associated with protection from re-infection in the field.^30^ Nevertheless, it is important to remember that the parasite itself escapes immunity by modulating immune responses.^31^ Thus, further investigations of memory B cells’ survival^32^ and dendritic cells’ functionality^33^ after infectious challenge would help to identify predictive factors of long-term efficacy in human.

To evaluate protection, we used C57BL/6 mice and *Pb*A model, which is a relevant model of liver stage immunity that closely resembles the situation in humans. In this model, sterile protection is not as easy as for Balb/c mice, where CS*Pb* is target of immuno-dominant and protective CD8+ T cell response.^34^ Indeed, CS seems to contain no naturally processed and presented H-2^b^ restricted epitopes.^24^ Sv129 hCD46IFNAR mice and C57BL/6 mice both expressed H-2^b^ major histocompatibility complex. We showed that they were similarly sensitive to *Pb*A challenge, with similar clinical features and no statistical difference in parasitemia on days 4, 5, and 6 post-infection. Palomo et al. showed a slightly delayed experimental cerebral malaria development and prolonged survival of C57BL/6 IFNAR mice, as compared to wild-type mice.^35^ Nevertheless, we defined ethical endpoints at the beginning of the study that have imposed an early sacrifice at day 6 or 7 post-infection, and we did not wait for natural death to avoid unnecessary suffering. We did not evaluate parasite burden in the liver but rather measured blood parasitemia, a more pertinent measurement since complete protection against blood infection is the final goal of a successful intervention. Given that CSP is not expressed in the blood stage, all the immune responses in our study have to act on sporozoites and liver stage. The reduction in blood stage parasitemia at days 4–6 post-challenge in vaccinated animals is a direct reflection of reduction in liver stage parasite densities. Neither measles virus (control) nor rMV-CSP immunization affect the exponential growth of parasites in the blood (lines are parallel in the log-transformed graphs, Figs. 2f, 3d, e), discarding unspecific effects of MV immunization or specific effect of anti-CSP immune response in the blood-stage growth. Importantly, we are measuring parasitemia with a resolution of 0.001% using a strong fluorescent parasite and liver load measurement is not as sensitive requiring the use of a higher number of parasites. Moreover, liver parasite burden does not necessarily reflect blood infection.^36^ So, the repetition of these experiments to measure liver stage parasites would not improve the analysis of this vaccine efficacy. rMV-CS*Pb* was able to elicit sterile protection in 33% of mice and to protect all of them from severe disease, with a reduced parasitemia, and no severe clinical symptoms. In the rGFP-*PbA*-CS*Pf* repeat challenge model, there was no sterile protection and reduction in parasitemia was less compared to the *Pb*A model. This suggests that sterile protection was not induced by neutralizing antibodies directed against the repeat sequence of CS*Pf*, but may involve antibodies against C and N-terminal domains of CS and cell-mediated immune responses. In fact, phagocytic activity of antibodies induced by RTS,S/AS01 malaria vaccine has been correlated with full-length CS and C-terminal-specific antibody titer, but not to repeat region antibody titer.^37^ Accordingly, we showed a moderate but significant induction of cell-mediated immune response that appeared as early as 7 days after a single immunization, with an increase in CD4^+^ and CD8^+^-specific T cells secreting IFNγ or TNFα. As there is no described CD8^+^ epitope for CS*Pf* in H-2^b^ mice, the increase observed, even if moderate, is of great interest. Indeed, protection against malaria has been correlated to CS*Pf* CD8^+^ T cell response in human immune system (HIS) mice harboring functional human CD8^+^ T cells.^38^ This major role for CD8^+^ T cells to induce protection was already shown by in vivo depletion of CD8^+^ T cells that abrogated sporozoïte-induced protective immunity in mice.^39^ Thus, even if the protection resulting from rGFP-*PbA*-CS*Pf* repeated challenge model was not indicative of real protection, it brought indications of efficient immune mechanisms involved in protection.

To conclude, in this work we demonstrated the promising potential of using measles vaccine vector to deliver malaria antigens. We showed the induction of cellular immune responses and long-term memory with high antibody titers in mice, two critical desired characteristics for second-generation malaria vaccines. As expected, expression of CS alone was not able to induce sterile protection in all mice in this model. We rather used CS as a “gold standard” to validate the measles vector approach. Transposition to clinical trial with measles vector is facilitated by an excellent track record of safety and immunogenicity for other relevant antigens and pathogens, and a well-established production process that allows substantial acceleration in development timelines. Further recombinant measles-vectored malaria vaccine candidates expressing additional pre-erythrocytic and/or blood-stage antigens in combination with CS will be soon evaluated to yield synergistic effects and provide protection with higher efficacy for long duration. Such vectors should be rapidly introduced in clinical trials and easily tested in controlled malaria human challenge, thanks to the excellent safety of measles vaccine.

## Methods

### Study design

This study aimed to evaluate the immunogenicity and efficacy of measles live attenuated vaccine as a vector to deliver malaria antigens. To this purpose, we designed, cloned, and rescued recombinant measles viruses (rMV) that expressed the CS of *Pb* ANKA (CS*Pb* ANKA full length sequence, mammalian codon optimized synthetic gene, Eurofins Genomics) or a truncated CS of *Pf* 3D7 (CS*Pf*, mammalian codon optimized synthetic gene, aa 19–369 without GPI anchored signal at C-terminus and with the signal sequence from MV Fusion protein at N-terminus; Genscript, USA). Rescue of both recombinant viruses (rMV-CS*Pb* and rMV-CS*Pf*) was performed using the helper-cell-based rescue method^22^ described by Radecke et al.^40,41^ and modified by Parks et al.^41^ (supplementary methods). rMV-CS*Pb* and rMV-CS*Pf* were grown on Vero cells (ATCC, CCL-81). Then, groups of six 6-week-old mice deficient for type-I IFN receptor (IFNAR) and expressing human CD46 (hCD46),^22^ housed under pathogen-free conditions at the Institut Pasteur animal facility, were inoculated with 10^5^ TCID_50_ of rMV-CS*Pb*, rMV-CS*Pf*, or MV-Schwarz as control, via the i.p. route. All the animal studies were repeated twice. To study cellular response, a single immunization was administered and spleens were extracted 7 days later. For humoral response and infectious challenge, two immunizations were administered within a 4 weeks interval. Sera were collected before the first immunization (day 0, negative control) and 4 weeks after each immunization, and 4 months after the second immunization to study long-term memory responses. Immunized mice were challenged with *Pb*A sporozoites expressing the green fluorescent protein (GFP) under the control of *hsp70* promoter (GFP-*Pb*A).^42^ Alternatively, mice immunized with rMV-CS*Pf* were challenged with *P. berghei* NK65 sporozoites expressing the GFP under the control of the *hsp70* promoter^43^ and a chimeric CS*Pb* harboring the central repetitive region of CS*Pf* (rGFP-*Pb*-CS*Pf* repeat).^44^ rGFP-*Pb-*CS*Pf* repeat parasites were generated by a genetic cross as described by Ishino et al.^42^ Sporozoites were freshly collected from the salivary gland of infected *Anopheles stephensi* in D-PBS and filtered using a 35 µm nylon mesh cell strainer snap cap (Corning Falcon). Infectious challenges were executed 4 weeks after the second immunization (early response), or 4 months after the second immunization (long-term memory response) by the microinjection of 5000 sporozoites in one microliter of D-PBS in the posterior footpad using a 35G microsyringe (World Precision Instruments). After challenge, parasitemia was monitored from day 3 to day 10. Blood samples (2 µl) were diluted in 500 µl of PBS and analyzed by flow cytometry (MacsQuant, Miltenyi Biotec). Doublets and clusters of red blood cells (RBCs) were excluded from counts. Single GFP+RBCs (infected RBC, iRBCs) among total RBCs were estimated and data analyzed by the MACSQuantify™ Software. As no protection against blood stage parasites was expected, mice were sacrificed at day 10 post-challenge in the presence of iRBCs in order to avoid unnecessary suffering, or before in the presence of severe symptoms that were ethical endpoints (signs of cerebral malaria: motor troubles, ruffled fur, and sometimes convulsions). Non-parasitemic mice at day 10 were considered sterile protected.

### Ethics statement

Experiments were conducted following the guidelines of the Office of Laboratory Animal Care at Institut Pasteur. The experimental protocol was approved by the Ethic Comity Ile-de-France—Paris 1 (no. 2014-061). All the experimenters had a regulatory authorization for animal handling delivered by the accredited French authorities and accepted by Institut Pasteur Animal Facility.

### Characterization of recombinant viruses

Virus growth curves were done on monolayers of Vero cells grown in 24-mm-diameter dishes (six-well plates) that were infected with rMV-CS*Pb* and rMV-CS*Pf* at an MOI of 1. At various times post-infection, cells were scraped into culture medium. After freeze thawing of cells and medium, and clarification of cell debris, virus titers were determined by endpoint dilution assay. For this purpose, Vero cells were seeded into 96-well plates (7500 cells/well) and infected with serial 1:10 dilutions of virus sample in DMEM-5% FCS. After incubation for 7 days, cells were stained with crystal violet, and the TCID_50_ values were calculated by use of the Spaerman–Kärber method.^45^ Expression of CS*Pf* and CS*Pb* was assessed in Vero cells infected with rMV-CS*Pb* and rMV-CS*Pf* by immunofluorescence assay (IFA) and Western blotting. IFA was performed on Vero cells at 36 h post-infection with rMV-CS*Pb* and rMV-CS*Pf* at an MOI of O.1. Cells were probed with 3D11 mouse anti-CS*Pb* monoclonal antibody (1/1000 dilution) (#MR4-100 hybridoma) or 2A10 mouse anti-CS*Pf* monoclonal antibody (1/1000 dilution) (#MR4-183 hybridoma). Cy3-conjugated goat anti-mouse IgG (Jackson immunoresearch laboratories) was used as secondary antibody (1/1000 dilution). Western blotting was performed on infected Vero cell lysates or supernatants fractionated by SDS–PAGE and transferred to cellulose membranes. 3D11 mouse anti-CS*Pb* monoclonal antibody and 2A10 mouse anti-CS*Pf* monoclonal antibody were used to detect CS proteins. A goat anti-mouse IgG-horseradish peroxidase (HRP) conjugate (#P0447, Dako) was used as secondary antibody.

### ELISA

MV antigen (Edmonston strain, #PR-BA102-S-L, Jena Bioscience) at 1 µg/ml in PBS, and CS*Pb* or CS*Pf* recombinant proteins (produced at the Recombinant Protein and Antibodies Production Core Facility of the Institut Pasteur by J. Bellalou and V. Bondet, using the BioPod F800 microfermentor battery) at 1 µg/ml in carbonate buffer were coated overnight at 4 °C onto 96-well plates (#439454, Thermo Scientific) and then blocked for 1 h at 37 °C with a saturation buffer (PBS, 0.05% Tween, 3% BSA). Sera samples from immunized mice were serially diluted (PBS, 0.05% Tween, 1% BSA) and incubated on plates for 1 h at 37 °C. After washing steps (0.05% Tween in PBS), a secondary HRP-conjugated goat anti-mouse Ig antibody (#115-035-146, Jackson ImmunoResearch) was added at a dilution of 1/1000 for 1 h at 37 °C. Antibody binding was revealed by addition of the TMB substrate (#5120-0047, Eurobio) and the reaction was stopped by addition of H_2_SO_4_ 1 M. The optical densities (O.D.) were recorded at 450 nm. The endpoint titers for each individual serum were calculated as the reciprocal of the last dilution giving twice the absorbance of negative control sera.

### ELISPOT assay

Freshly extracted splenocytes from immunized mice were tested for their capacity to secrete IFN-γ upon specific stimulation. Multiscreen-HA 96-well plates (#MSIP4510, Millipore) were coated overnight at 4 °C with 5 µg/ml of anti-mouse IFN-γ (#551216, BD Biosciences Pharmingen) in PBS and, after washing, were blocked for 2 h at 37 °C with complete MEM (MEM—10% FCS supplemented with non-essential amino-acids 1%, sodium pyruvate 1%, and β-mercapto-ethanol). The medium was then replaced with 100 µl of cell suspension containing 2 × 10^5^ splenocytes in each well (triplicate) and 100 µl of stimulating agent in complete MEM. Plates were incubated for 40 h at 37 °C. Cells were stimulated with Concanavalin A (#C-5275, Sigma) as positive control, complete MEM as negative control, live attenuated MV-Schwarz virus at an MOI of 1, and CS*Pf* recombinant protein at 50 µg/ml. After incubation and washing, biotinylated anti-mouse IFN-γ antibody (#554410, BD Biosciences Pharmingen) was added at a dilution of 1/500 and plates were incubated for 120 min at room temperature. After extensive washing, streptavidin–alkaline phosphatase conjugate (#7100-05, Clinisciences) was added at a dilution of 1/1000 and plates were incubated 1 h at room temperature. Spots were developed with BCIP/NBT (#B1911, Sigma) and counted in an ELISPOT reader (CTL ImmunoSpot®).

### Intracellular cytokine staining

Freshly extracted splenocytes from immunized mice were analyzed by flow cytometry for their capacity to secrete IFN-γ and TNF-α upon specific stimulation. Spleen cells were cultured for 16 h in U-bottom 96-well plates (1.0 × 10^6^ cells/well) in a volume of 0.2 ml complete medium (MEM—10% FCS supplemented with non-essential amino-acids 1%, sodium pyruvate 1%, and β-mercapto-ethanol). Cells were stimulated with PMA/ionomycin (#00-4970, ebioscience) as positive control, complete MEM as negative control, live attenuated MV-Schwarz virus at an MOI of 1, and CS*Pf* LPS-free recombinant protein at 50 µg/ml. Brefeldin A (#B6542, Sigma) was then added at 10 µg/ml for 6 more hours of incubation. Stimulated cells were harvested, washed in phosphate-buffered saline containing 1% bovine serum albumin and 0.1% w/w sodium azide (FACS buffer), incubated 10 min with Fc blocking Ab (CD16/32 clone 2.4G2, PharMingen) and surface stained in FACS buffer with Live/Dead fixable dead cell violet kit (#L34955, invitrogen), anti-mouse CD4-PECy7 mAb (#552775, BD Biosciences) and anti-mouse CD8-APCH7 mAb (#560182, BD Biosciences) for 30 min at 4 °C in the dark. After washing, cells were fixed and permeabilised for intracellular cytokine staining using the Cytofix/Cytoperm kit (#554922, BD Bioscience). Cells were then incubated in a mix of anti-mouse IFNγ-APC mAb (#554413, BD Biosciences) and anti-mouse TNF-α-FITC mAb (#554418, BD Biosciences) diluted in permwash buffer (# 557885, BD Bioscience) for 30 min in the dark. After washing with permwash buffer and FACS buffer, cells were fixed with 1% formaldehyde in PBS. Data were acquired using a MacsQuant® Analyzer (Miltenyi Biotec), and analyzed using Flow Jo^TM^ 9.3.2 software and are presented as percentage of CD4^+^ or CD8^+^ cells expressing TNF-α or IFNγ among total CD4 or CD8 populations.

### Statistical analysis

Parasitemia was log transformed for normalization. Statistical analyses of normalized parasitemia were done using the *t*-test. Statistical analyses of cellular responses were done using the non-parametric Mann–Whitney *U*-test. Differences were considered statistically significant when *p* < 0.05.

### Reporting summary

Further information on experimental design is available in the Nature Research Reporting Summary linked to this article.

## Supplementary information

Reporting Summary

Supplemental Material

## Data Availability

The data that support the findings of this study are available from the authors on reasonable request; see author contributions for specific data sets.

## Supplementary information

**Supplementary information** accompanies the paper on the *npj Vaccines* website (10.1038/s41541-019-0106-8).
