# Supplementary material for: Recombinant measles vaccine expressing malaria antigens induces long-term memory and protection in mice
Source: NPJ Vaccines. 2019 Feb 27;4:12. doi: 10.1038/s41541-019-0106-8 (PMC6393439; doi:10.1038/s41541-019-0106-8)
Supplement: Supplementary file 2 — Supplemental Material [file 41541_2019_106_MOESM2_ESM.pdf]

Supplementary information

Supplementary methods

Construction of pTM2-MVSchw-CS*Pb* and pTM2-MVSchw-CS*Pf* plasmids and rescue of rMV-CS*Pb* and rMV-CS*Pf* recombinant viruses.

The plasmid pTM2-Schw encodes the cDNA of the anti-genome of the Schwarz MV vaccine strain with an additional transcription unit (ATU) between the phosphoprotein (MV-P) and the matrix (MV-M) genes, flanked by BsiWI/BssHII restriction sites. Two cDNAs encoding the circumsporozoïte protein of *Plasmodium berghei* ANKA (CSPb ANKA full length sequence, mammalian codon optimized synthetic gene, Eurofins Genomics) and the circumsporozoïte protein of *Plasmodium falciparum* 3D7 (CSPf, truncated form from 19 to 369 aa, without GPI anchored signal at C-terminus, signal sequence from MV Fusion protein at N-terminus, chemically synthesized; Genscript, USA) were inserted in ATU2, to produce respectively pTM2-MVSchw-CS*Pb* and pTM2-MVSchw-CS*Pf* plasmids. The sequences, which were codon optimized for expression in mammalian cells, respected the "rule of six", which stipulates that the number of nucleotides in the MV genome must be a multiple of 6, and contain BsiWI/BssHII restriction sites at both ends.

HEK293-T7-MV helper cells stably express T7 polymerase and MV-N and MV-P proteins and are used for measles viral rescue (Tangy, EU patent, 2006). HEK293-T7-MV helper cells were transfected by using the calcium phosphate procedure with pTM-MVSchw (5 µg) and a plasmid expressing the MV polymerase L gene (pEMC-La; 20 ng; a kind gift of M. A. Billeter). After overnight incubation at 37°C, the transfection medium was replaced by fresh medium and the cells were heat shocked at 42,5°C for 3 h and then returned to 37°C. After 2 days of incubation at 37°C, transfected cells were transferred onto a monolayer of Vero cells (ATCC, CCL-81) and incubated at 37°C. Single syncytia were transferred to 35-mm wells of Vero cells and then were expanded to larger dishes. Viruses were harvested when syncytia involved 80 to 90% of the culture (usually after 2 days) by scraping infected cells, freeze-thawing cells and medium, and centrifuging them to remove cellular debris.

Supplementary Figures

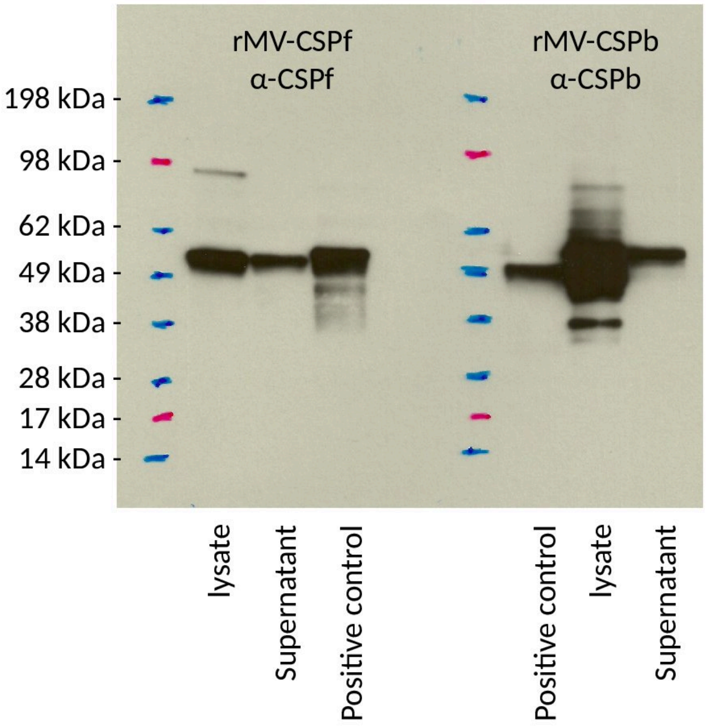

Supplementary Figure 1: Full, uncropped blot from Figure 1c with molecular weigh marker.

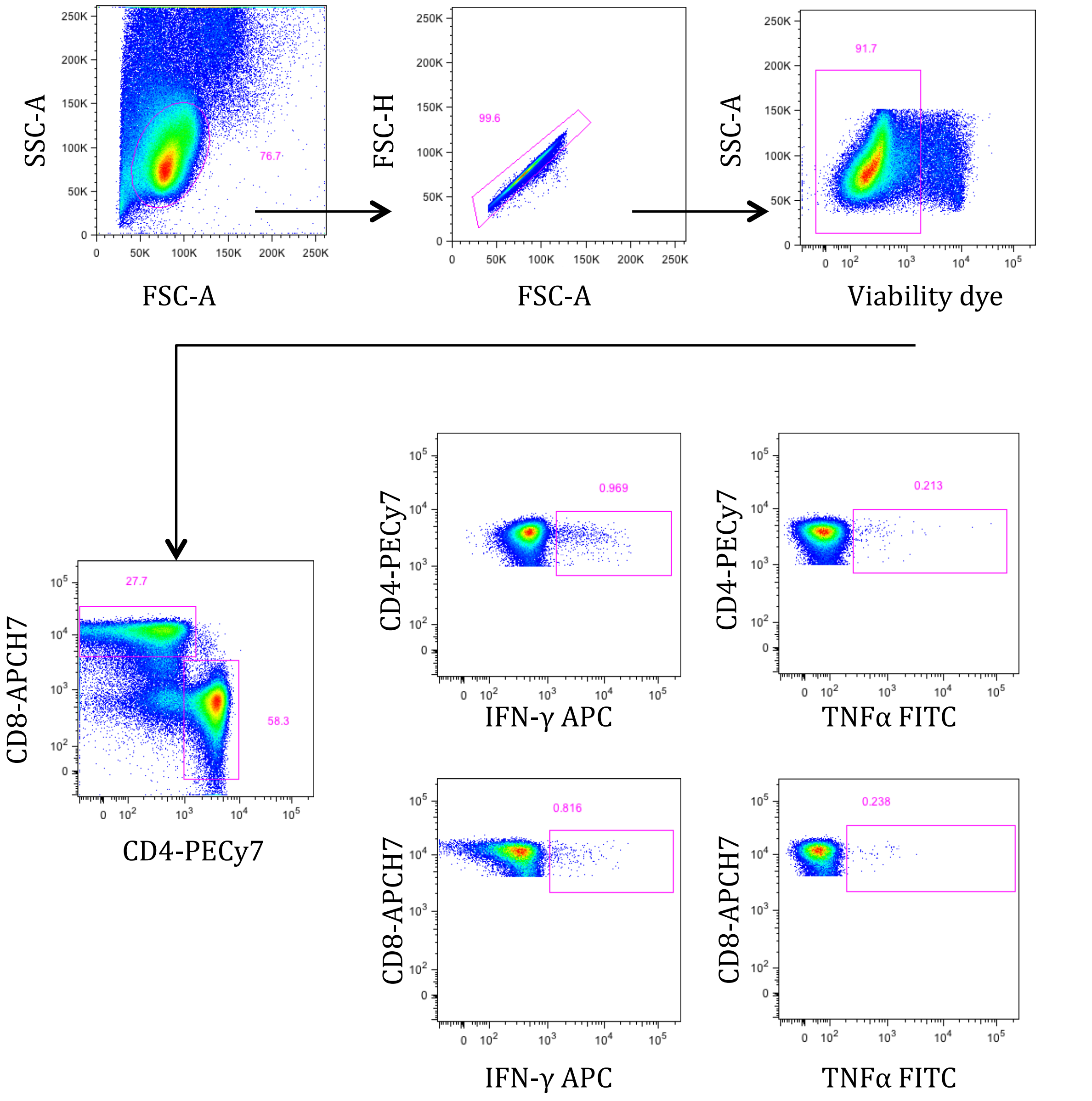

Supplementary Figure 2: Gating strategy used for Figure 4b-e. Single live lymphocytes were gating using successively FSC-A/SSC-A, FSC-A/FSC-H and a viability dye. Then CD4+ and CD8+ T cells were separated in order to measure the percentage or IFN-γ and TNF-α secreting cells in each groups.
